# Low dose Naltrexone for induction of remission in inflammatory bowel disease patients

**DOI:** 10.1186/s12967-018-1427-5

**Published:** 2018-03-09

**Authors:** Mitchell R. K. L. Lie, Janine van der Giessen, Gwenny M. Fuhler, Alison de Lima, Maikel P. Peppelenbosch, Cokkie van der Ent, C. Janneke van der Woude

**Affiliations:** Department of Gastroenterology and Hepatology, Erasmus MC-University Medical Centre Rotterdam, s Gravendijkwal 230, 3015 CE Rotterdam, The Netherlands

**Keywords:** Crohn’s disease, Ulcerative colitis, Naltrexone, Endoplasmic reticulum stress, Wound healing

## Abstract

**Background:**

Around 30% of patients with inflammatory bowel disease (IBD) are refractory to current IBD drugs or relapse over time. Novel treatments are called for, and low dose Naltrexone (LDN) may provide a safe, easily accessible alternative treatment option for these patients. We investigated the potential of LDN to induce clinical response in therapy refractory IBD patients, and investigated its direct effects on epithelial barrier function.

**Methods:**

Patients not in remission and not responding to conventional therapy were offered to initiate LDN as a concomitant treatment. In total 47 IBD patients prescribed LDN were followed prospectively for 12 weeks. Where available, endoscopic remission data, serum and biopsies were collected. Further the effect of Naltrexone on wound healing (scratch assay), cytokine production and endoplasmic reticulum (ER) stress (GRP78 and CHOP western blot analysis, immunohistochemistry) were investigated in HCT116 and CACO2 intestinal epithelial cells, human IBD intestinal organoids and patient samples.

**Results:**

Low dose Naltrexone induced clinical improvement in 74.5%, and remission in 25.5% of patients. Naltrexone improved wound healing and reduced ER stress induced by Tunicamycin, lipopolysaccharide or bacteria in epithelial barriers. Inflamed mucosa from IBD patients showed high ER stress levels, which was reduced in patients treated with LDN. Cytokine levels in neither epithelial cells nor serum from IBD patients were affected.

**Conclusions:**

Naltrexone directly improves epithelial barrier function by improving wound healing and reducing mucosal ER stress levels. Low dose Naltrexone treatment is effective and safe, and could be considered for the treatment of therapy refractory IBD patients.

**Electronic supplementary material:**

The online version of this article (10.1186/s12967-018-1427-5) contains supplementary material, which is available to authorized users.

## Background

Inflammatory bowel disease (IBD) is a chronic inflammatory disorder, which includes Crohn’s disease (CD) and ulcerative colitis (UC). The aim of therapy is to induce sustained remission, a state of long lasting quiescent disease. Several drugs exist to induce and maintain remission, and these drugs are usually prescribed in a step-up fashion. Nevertheless, even when using this step-up strategy, a subset of patients will fail to reach or maintain remission even with the most potent therapies, often due to drug intolerance or loss of efficacy. For instance, yearly loss of efficacy rates are 13–24% in patients treated with anti-tumor necrosis factor α (anti-TNFα) agents [1, 2]. In general, such a state of therapy refractoriness occurs in 35–60% of all IBD patients and severely limits treatment options, often resulting in surgery or corticosteroid dependency [3, 4]. Thus, for this subset of patients, alternative treatments remain of continued interest.

The etiology of IBD is complex, with genetic predisposition, an altered microbiome, environmental factors and a weakened epithelial barrier function triggering a chronic mucosal immune response. Targeting these different causes with medication is the current challenge in IBD treatment. Previous research suggests that the endogenous opioid system also plays a role in gut immunity [5, 6]. For instance, in IBD patients, the µ-opioid receptor (MOR) is overexpressed in mucosal T-lymphocytes and monocytes, and ex vivo stimulation of MOR with the agonist DALDA reduced TNFα in mucosal biopsies from IBD patients [7]. In addition, DALDA also showed anti-inflammatory responses in a mouse model of colitis through inhibition of T cell proliferation and cytokine (including TNFα) production [8]. Another opioid known to modulate MOR responses is Naltrexone. While being a MOR *anta*gonist, which blocks endogenous opioid effects when used at high concentrations [9], administration of low dose Naltrexone (LDN) is postulated to result in upregulation of endogenous encephalin and endorphin levels and to have a positive modulatory effect on the MOR [10, 11]. Thus, the use of LDN in clinical settings is gaining interest, with Crohn’s disease, multiple sclerosis and fibromyalgia described as potential targets for treatment with LDN [12–14]. In both mouse and rat models of IBD, LDN alleviated inflammation, in part by reducing pro-inflammatory cytokine production [15, 16]. Interestingly, we and others have shown that endoplasmic reticulum (ER) stress in intestinal Paneth cells is one of the contributing factors in IBD [17–19], and it was recently reported that Naltrexone attenuates inflammation in a mouse liver injury model by reducing ER stress [20, 21].

Pilot studies in patients with CD showed a positive effect of LDN therapy, with 15 of 17 patients showing a clinical response [22]. A subsequent randomized, placebo-controlled, double blind study in 34 patients found a response rate of 88% in the LDN group versus 40% in the placebo group after 12 weeks of therapy [23]. In addition LDN was also shown to be safe in pediatric IBD patients, and resulted in significantly reduced PCDAI scores, with 25% of patients achieving remission and 67% showing improvement of disease [24]. The above results suggest that LDN is an effective therapy for CD patients, although the exact mechanism remains unclear. Based on these promising results, patients started to request LDN therapy due to its favorable side-effect profile, and at the Erasmus MC we decided to start prescribing LDN to therapy refractory IBD patients with active disease. The aims of this study were to assess the clinical effect of LDN and to investigate whether LDN has a direct modulatory effect on intestinal epithelial barrier function.

## Methods

### Clinical cohort

A prospective cohort of patients with therapy refractory IBD (CD or UC) that started LDN therapy was formed. The decision to start LDN therapy was made by the treating physician, after fully informing the patient of the possible benefits and drawbacks. All patients were prescribed 4.5 mg Naltrexone once daily. Patients were instructed to administer one dose of LDN before bedtime.

Upon initiation of LDN therapy, patients were followed according to usual care at the outpatient clinic, with contact (in person or via telephone) after 4, 8 and 12 weeks. During these visits self-assessed disease activity and adverse events were recorded. Patients were offered endoscopic evaluation and assessment of laboratory values after 12 weeks of treatment or at time of discontinuation of LDN therapy, whichever occurred earlier. This study was approved by the Ethical Committee of the Erasmus MC (MEC-2014-656).

### Clinical data collection

During the follow-up, demographic data (e.g. age, gender) and IBD related data (e.g. year of diagnosis, concomitant and previous IBD related therapies, Montreal phenotype classification) were recorded. Additionally, where available, data on diagnostic tests, particularly endoscopic evaluations, performed prior to the start of LDN therapy (with a 1 week window) and during the follow-up period were recorded. All patients that completed at least 1 assessment of disease activity were included in the cohort.

### Clinical outcome measures

Clinical outcomes were based on patient self-assessments and outpatient assessments, where available. Patients were considered non-responders if no clinical improvement occurred in the first 4 weeks of LDN therapy. Patients were considered to have clinical response if self-assessed disease activity decreased within the first 4 weeks of LDN therapy, and lasted for at least 4 weeks in total. Of secondary interest were the rates of adverse events during LDN therapy. Endoscopy results were scored based on the most severe area of inflammation. Endoscopic findings in all IBD patients were scored on a scale from 0 to 3, representing no inflammation to severe inflammation respectively.

### Cell lines

Colorectal cancer cell lines HCT116 and CACO-2 were cultured in Dulbecco’s Modified Eagles Medium (DMEM, Lonza, Basel, Switzerland) supplemented with 100 U/mL penicillin, 100 mg/mL streptomycin (Life technologies, Bleiswijk, NL) and 10% Fecal Calf Serum (FCS, Sigma-Aldrich, St. Louis, USA). Cells were maintained at 37 °C in a 5% CO_2_ humidified setting.

### Organoid culture

Non-inflamed intestinal biopsies were collected from two IBD patients undergoing routine endoscopy for their disease. Organoids were prepared as described [25, 26], see Additional file 1 for details.

### Cell viability assay

Cell viability was assessed using MTT assays as described [27], see Additional file 1: Methods. Each experiment was performed twice in triplicate.

### Wound healing assay

Wound healing assays were performed as described [28], see Additional file 1. The concentration of Naltrexone used was based on in vivo dosages (4.5 mg per ± 60 kg bodyweight). Experiments were performed thrice in duplicate, with two measure-sites per scratch.

### Western blotting

Western blotting was performed as described [29], with modifications (see Additional file 1). HCT116 and CACO-2 cells were treated with Tunicamycin (2 μM), lipopolysaccharide (10 μg/mL LPS) or *E. coli* (paraffin-fixed DH5α, 6.25e5/mL) in the presence or absence of 1 μg/mL Naltrexone. Organoids were treated with LPS in the presence or absence of 1 μg/mL Naltrexone. Experiments were performed at least twice.

### Immunohistochemistry

FFPE tissue sections were immunohistologically stained for GRP78, as described [17, 30], see Additional file 1. Antigen retrieval was performed by boiling the slides in 600 mL of 10 mM sodium citrate buffer, pH 6.0 for 15 min. Slides were blocked by incubating in 10% goat serum in PBS and incubated with GRP78 antibody (BiP, Cell Signaling Technology, Danvers, MA) diluted in blocking buffer (1:100) overnight at 4 °C. Rabbit envision (DAKO, Heverlee, Belgium) was used as secondary antibody.

### Reverse transcriptase polymerase chain reaction (rt-PCR)

We used rt-PCR to determine MOR expression on the IEC cell lines, using Ribosomal protein (*RP2*) primers were used as control [26], see Additional file 1.

### Enzyme linked immunosorbent assay (ELISA)

Cells were plated at 0.2 × 10^6^ per well in 24 wells plates. Upon attachment to the plate, cells were treated as described in the text and supernatant was harvested after 24 h. Experiments were performed twice, in duplicate. Cytokine levels in supernatants from IECs and patient sera were determined by ELISA (Ready-SET-Go!^®^ eBioscience, San Diego, CA) as per manufacturer’s instructions. All samples were tested in duplicate in the ELISAs.

### Statistical analysis

Continuous variables were reported as medians with interquartile range (IQR). Comparisons in continuous variables were performed with the Mann–Whitney U test. For comparisons of categorical variables, Fisher’s exact test was used. For in vitro and ex vivo experiments, normality of distribution was assessed with D’agostino and Pearson Omnibus normality test. When passing normality test or when there were insufficient numbers to calculate normality, parametric testing was performed, otherwise, non-parametric tests were employed. Student T-tests were performed for comparisons of two groups. For comparisons of more than two groups, ANOVA with post hoc testing (Tukey’s multiple comparison test) was performed. For all tests, one or two-sided (as appropriate) p-values < 0.05 were considered statistically significant, graphs show mean ± SEM. Analyses were performed using Graphpad Prism 5.0.

## Results

### Patient characteristics

From July 2010 till August 2014, 47 patients were treated with LDN, of which 19 (40.4%) were male and 28 were female. Median treatment and follow-up duration after start of LDN was 3 months (IQR 3–5 months). Of the 47 patients, 28 (59.6%) were diagnosed with CD and 19 with UC. Three patients had previously undergone surgery (2 ileocecal resections and 1 subtotal colectomy, all in CD patients). The full baseline patient characteristics are described in Table 1.

**Table 1:** Baseline patient characteristics

General characteristics					
Diagnosis, N (%)	UC, 19 (40%)		CD, 28 (60%)		Combined, 47
Gender (M/F)	10/9		9/19		19/28
Median age at diagnosis (years, IQR)	31 (27–44.5)		23 (16.8–32.5)		27 (18–39.5)
Median age at start of LDN (years, IQR)	42 (33.5–52)		35.5 (25.5–53.5)		40 (27.5–52.5)
Median disease duration at start of LDN (years, IQR)	6.9 (3.2–12.4)		7.8 (3.8–16.5)		7.0 (3.8–13.4)
CRP mg/L, median (IQR)	7 (2–27)		6 (2–7)		6 (2–9)
Endoscopic score (median, IQR)	2.0 (1.0–2.0)		2.0 (2.0–2.0)		2.0 (1.25–2.0)
Disease characteristics					
Disease extent or phenotype [montreal classification, % (N)]	E1	11% (2)	L1	7% (2)	
	E2	63% (12)	L2	32% (9)	
	E3	26% (5)	L3	61% (17)	

*CD* Crohn’s disease, *CRP* C-reactive protein, *IQR* inter-quartile range, *LDN* low dose Naltrexone, *UC* ulcerative colitis

All participants were either steroid dependent or steroid refractory and had previously been treated with at least one other drug, and all patients showed clinical signs of disease activity at initiation of LDN therapy. Notably, 41 patients (87.2%) had previously received at least one anti-TNFα agent, and 19 (40.4%) had been treated with two anti-TNFα agents. The 6 patients not exposed to anti-TNFα had refused anti-TNFα therapy due to fear of possible side effects. The full details on the previous and concomitant treatments at start of LDN therapy are described in Table 2. Seven patients (14.9%) reported adverse events due to LDN, including vivid dreams (N = 4), drowsiness (N = 2) and headache (N = 1). Two patients discontinued LDN therapy after 2 weeks, due to drowsiness. Vivid dream complaints improved when LDN was administered in the morning instead of at bedtime.

**Table 2:** Medical therapies used prior to start of LDN and concomitantly with LDN

	UC, 19	CD, 28	Combined, 47
Prior and concomitant therapies			
Therapies prior to LDN, N (%)			
5-ASA	17 (89%)	14 (50%)	31 (66%)
Steroids	19 (100%)	28 (100%)	47 (100%)
Immunosuppressives	18 (95%)	27 (96%)	45 (96%)
Anti-TNF	16 (84%)	25 (89%)	41 (87%)
Other	5 (26%)	2 (7%)	8 (15%)
Concomitant therapies at start of LDN, N (%)			
5-ASA	7 (37%)	3 (11%)	10 (21%)
Steroids	6 (32%)	18 (64%)	24 (51%)
Immunosuppressives	8 (42%)	9 (32%)	17 (36%)
Anti-TNF	5 (26%)	3 (11%)	8 (17%)
Other	1 (5%)	2 (7%)	3 (6%)
None	4 (21%)	7 (25%)	11 (23%)

Steroids refer to any form of corticosteroids. Immunosuppressives refer to thiopurines or methotrexate. Other refers to tacrolimus, cyclosporine, thioguanine or blinded trial drugs

*anti-TNF* anti-tumor necrosis factor, *CD* Crohn’s disease, *LDN* low dose Naltrexone, *UC* ulcerative colitis

### LDN therapy shows clinical and endoscopic efficacy in IBD patients

Of the 47 patients, 35 (74.5%) achieved a clinical response. Of those 35 patients, 12 patients had a response of at least 3 months (25.5% of total cohort, 8 CD, 4 UC), whereas a short-lived (between 4 and 12 weeks) improvement was seen in the remaining 23 patients (48.9% of total cohort, 13 CD, 10 UC). There was no statistically significant difference between CD and UC patients in the number of patients that achieved either response or remission (p = 1.000 and p = 0.515 respectively).

**Fig. 1:**
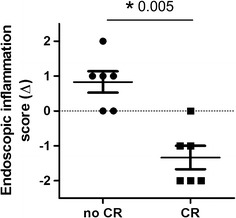
Changes in individual endoscopic inflammation scores values. The change in endoscopic score value for patients in clinical remission after week 12 and patients not in clinical remission after week 12 are displayed. Each dot represents an individual. The horizontal line represents the group median. The difference in group medians is statistically significant (− 1.5 versus 1.0, for clinical remission and not in clinical remission respectively, p = 0.005)

The median endoscopic score at baseline amongst all patients was 2 (IQR 1.25–2.0). In 12 patients, consecutive endoscopies were performed both at baseline and at 12 weeks or at time of relapse, whichever occurred earlier. These consecutive endoscopies were performed in 6 patients with response (3 CD, 3 UC) and 6 patients with remission (1 CD, 5 UC). Between these two groups, no significant difference was observed in baseline endoscopic score (median 1.67, range 1–3 versus 1.83, range 0–3 for response and remission respectively p = 0.676). However, patients achieving clinical remission had a significantly greater improvement in endoscopic score compared to patients not reaching clinical response (median change − 1.5, range − 2 to 0 versus 1.0, range 0–2, p = 0.005, Fig. 1). Complete endoscopic remission upon treatment with LDN was seen in 5 out of 6 patients with clinical remission.

### Naltrexone improves wound healing in intestinal epithelial cell layers

**Fig. 2:**
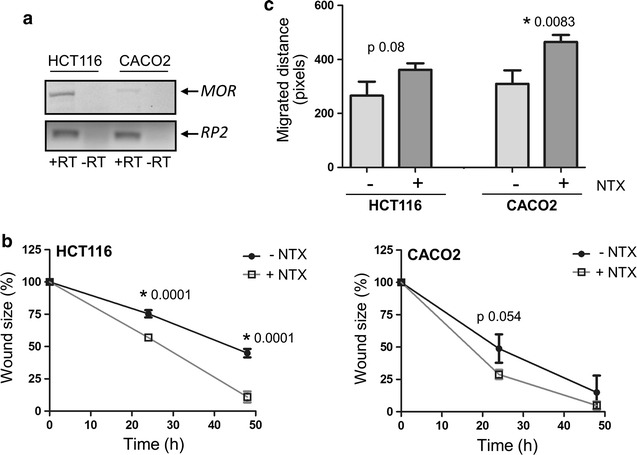
Naltrexone improves epithelial wound healing. **a** Expression of the μ-opioid receptor (MOR) was tested by rt-PCR, using -RT (i.e. RNA) controls. Ribosomal protein 2 (RP2) was used as cDNA quality control. **b** HCT116 (left panel) and CACO2 (right panel) cultures were scratched in the presence or absence of 1 μM Naltrexone (NTX), and wounds were photographed at t = 0, 24 and 48. Mean percentage wound size of three independent experiments is shown. **c** Migration of wound edges at t = 24 in pixels presented as mean of three experiments

Having shown clinical effect of Naltrexone in patients, we next sought to establish whether Naltrexone has a direct effect on epithelial cell function. After testing for the presence of MOR (Fig. 2a), we investigated the effect of Naltrexone on wound healing in layers of HCT116 and CACO2 colonic epithelial cell lines. Figure 2b shows that scratch wounds inflicted in HCT116 cell cultures are healed significantly faster when cells are treated with Naltrexone as compared to vehicle control (p = 0.0001 at t = 24; p = 0.0001 at t = 48). In CACO2 cells, which migrate much faster than HC116, all wounds were healed at t = 48 and the effect of Naltrexone on wound size was less clear (t = 24 h, p = 0.085, Fig. 2b, right panel). Possibly, the lower MOR expression levels observed in CACO2 cells accounts for the lesser effect of Naltrexone in this cell line. However, when comparing the migrated distance of cells, it was evident that Naltrexone stimulated epithelial cell migration, in both HCT116 (361 ± 24 vs 656 ± 52 pixels, p = 0.085) and CACO2 (465 ± 26 vs 310 ± 50 pixels, p = 0.0083, Fig. 2c). This effect of Naltrexone on wound healing was not due to an increased cellular proliferation, as total viable cell numbers were not affected by Naltrexone up to a concentration of 100 μg/mL (Additional file 2: Figure S1A–C).

### Naltrexone does not affect interleukin 8 (IL-8) cytokine levels in epithelial cells and patient sera

**Fig. 3:**
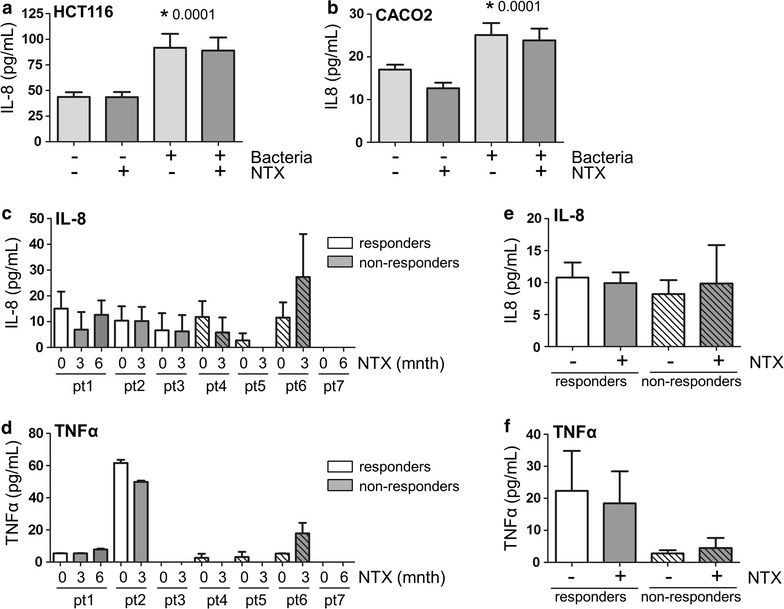
Naltrexone does not affect IL-8 levels in epithelial cell lines and patient sera. Stimulation of HCT116 (**a**) and CACO2 (**b**) cell layers for 24 h with bacteria results in significantly increased IL-8 levels in culture supernatants as determined by ELISA. Co-treatment with 1 μg/mL Naltrexone does not affect basal levels or bacteria-induced levels of IL-8 production in these cell lines. **c**–**f** Serum from patients was taken before low dose Naltrexone (NTX) treatment, and 3 or 6 months into treatment. IL-8 was detectable in 6 of 7 patients **c** by ELISA, whereas TNFα was detectable in 5 patients (**d**). There was no significant difference in the mean IL-8 (**e**) or TNFα (**f**) levels between responders and non-responders to low dose Naltrexone

In vivo, epithelial cells produce an array of cytokines in response to inflammatory stimuli, which in turn can attract immune cells and perpetuate inflammation in IBD patients. We therefore investigated whether cytokine production by epithelial cells is directly affected by Naltrexone. Cells were stimulated with bacteria in the absence or presence of Naltrexone, and supernatants were tested for the presence of the pro-inflammatory cytokines IL-6, IL-8 and TNFα after 24 h. As shown in Fig. 3, treatment of cells with bacteria significantly increased IL-8 production in both HCT116 and CACO2 cells (44 ± 4 to 92 ± 14 pg/mL in HCT116, p = 0.0001, Fig. 3a and 17 ± 1 to 25 ± 3 pg/mL in CACO2, p = 0.0001, Fig. 3b). However, neither basal levels nor bacteria-stimulated levels of IL-8 were significantly affected by Naltrexone treatment. IL-6 and TNFα levels were undetectable (not shown).

Next we tested whether IL-8 or TNFα systemic levels in patients were modulated by Naltrexone treatment in vivo. Paired serum samples (before and after initiation of treatment) were available of 7 patients, 3 responders and 4 non-responders. IL-8 was detected in 6 patients (Fig. 3c), whereas TNFα could be measured in serum from 5 out of 7 patients (Fig. 3d). No consistent up or down modulation of either IL-8 or TNFα was observed, nor were there significant differences in these cytokine levels between responders and non-responders to Naltrexone (Fig. 3e, f). Together, these data suggest that Naltrexone does not positively impact on inflammation through modulation of intestinal epithelial cell cytokine production.

### ER stress is intestinal epithelium is reduced by Naltrexone

**Fig. 4:**
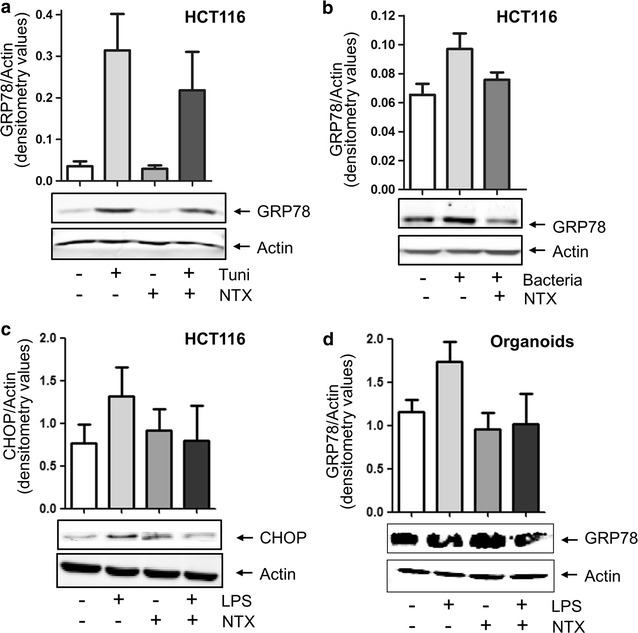
ER stress in epithelial cell lines is decreased by Naltrexone. **a** ER stress was induced in HCT116 cells by treatment with 2 μM Tunicamycin (Tuni), resulting in an upregulation of GRP78 expression levels as detected by Western Blot analysis. Co-treatment of cells with 1 μg/mL Naltrexone (NTX) reduces the amount of Tunicamycin-induced GRP78 expression. Upper graph: mean densitometry values of two independent experiments, GRP78 expression is corrected for Actin, to control for equal loading. Representative example is shown in the bottom panels. **b** Treatment of HCT116 cells with bacteria results in a significant upregulation of GRP78 expression as detected by Western blot analysis, which is reduced by co-treatment cells by treatment of cells with 1 μg/mL Naltrexone. Mean densitometry values of four independent experiments is shown. **c** Treatment of HCT116 cells with LPS results in a significant upregulation of CHOP expression as detected by Western blot analysis, which is reduced by co-treatment cells by treatment of cells with 1 μg/mL Naltrexone. Mean densitometry values of three independent experiments is shown. **d** Treatment of organoids with LPS results in a significant upregulation of GRP78 expression as detected by Western blot analysis, which is reduced by co-treatment cells by treatment of cells with 1 μg/mL Naltrexone. Mean densitometry values are shown of experiments performed on organoids derived from two individual donors, with two independent experiments each

As ER stress in the mucosa has been associated with the development of IBD, and Naltrexone was previously shown to reduce ER stress-induced inflammation in a model of liver damage, we next investigated whether Naltrexone has a direct effect on ER stress in intestinal epithelium. ER stress was chemically induced in intestinal epithelial cell lines by Tunicamycin (Fig. 4a and Additional file 3: Figure S2), as demonstrated by a strong upregulation of the ER stress marker GRP78. Interestingly, Naltrexone was able to reduce these levels in both cell lines. Chemical stimulation of cells with Tunicamycin causes inhibition of the UDP-*N*-acetylglucosamine-dolichol phosphate *N*-acetylglucosamine-1-phosphate transferase (GPT) and subsequent accumulation of unfolded glycoproteins in the ER; a non-physiological process likely to result in much higher ER stress levels than are probable in vivo. To investigate ER stress in a more physiologically relevant setting, we incubated intestinal epithelial cells with bacteria (representative examples shown in Fig. 4b and Additional file 3: Figure S2A). A significant upregulation of GRP78 expression was observed in HCT116 cells treated with *E. coli* (0.065 ± 0.007 to 0.097 ± 0.01, p = 0.0025, Fig. 4b), which again was significantly reduced by co-treatment of cells with Naltrexone (0.076 ± 0.005, p = 0.0025). In CACO2 cells, Naltrexone diminished bacteria-induced GRP78 expression in three out of three experiments, although this did not reach statistical significance as bacteria-induced ER stress was low in these cells (Additional file 3: Figure S2A). In order to further confirm ER stress pathway activation with a different physiological stimulus, we also induced ER stress in HCT116 cells with lipopolysaccharide and investigated expression levels of CHOP, a downstream target of the ER stress pathway. Figure 4c shows that CHOP levels induced by LPS were reduced by co-treatment with Naltrexone (1.315 ± 0.592 to 0.801 ± 0.710, p = 0.027). Again, the effect was less clear for CACO2 cells (Additional file 3: Figure S2C).

While cell lines are an easy and common model system to study epithelial cell function, such cell lines may show different cellular effects due to transforming mutations. We therefore generated organoids from colonic biopsies from two IBD patients, representing IBD epithelial tissue. Stimulation of these organoids with LPS again induced GRP78 expression (4 out of 4 experiments), and ER stress levels were reduced by co-treatment of organoids with Naltrexone (Fig. 4d, 1.734 ± 0.473 to 1.017 ± 0.698, p = 0.046). Together, these data imply that ER stress in intestinal epithelial cells is alleviated by Naltrexone.

**Fig. 5:**
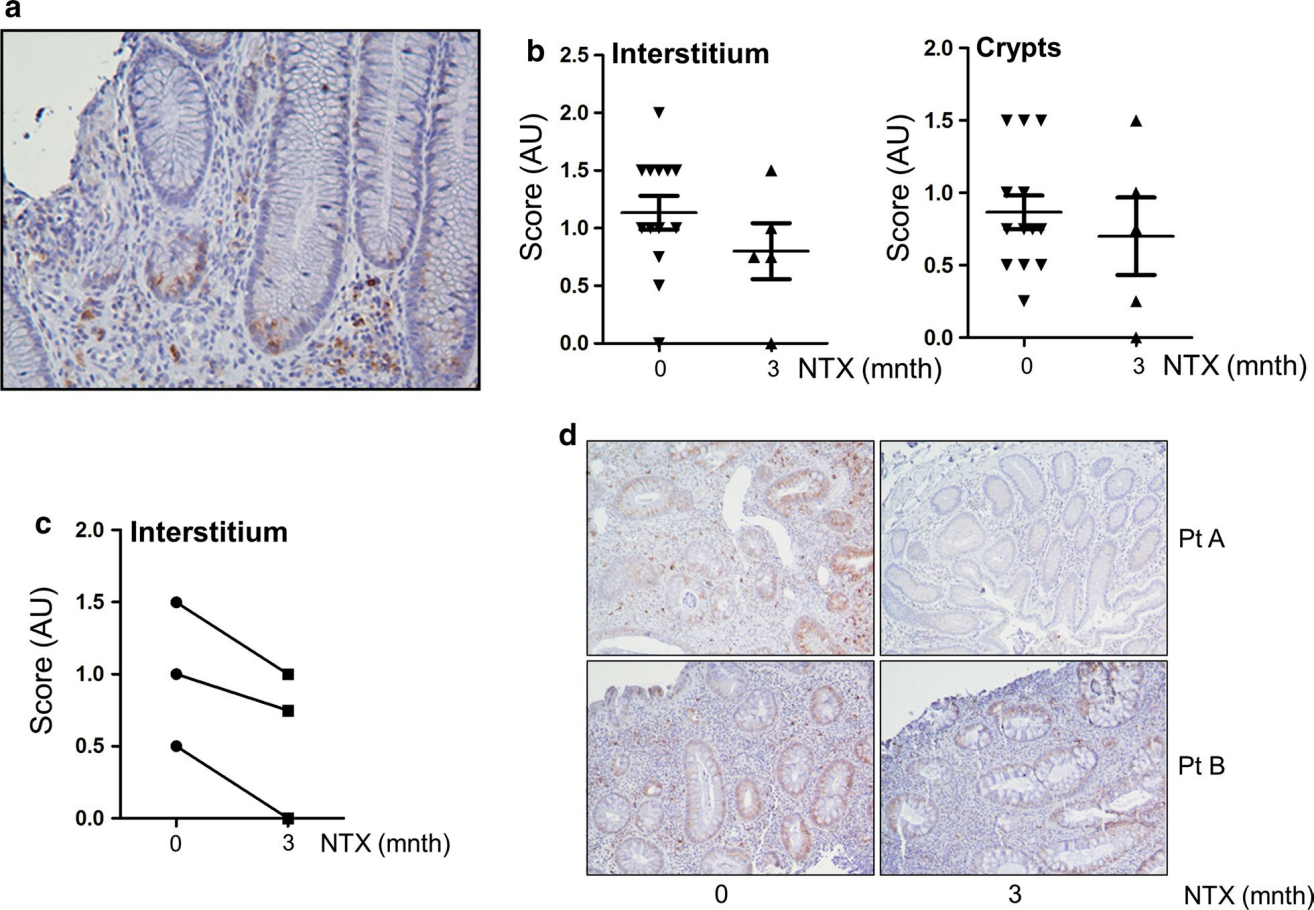
High ER stress in mucosa from IBD patients is reduced by low dose Naltrexone treatment. **a** Example of GRP78, showing high ER stress marker expression in crypts as well as lamina propria. **b** GRP78 intensity was scored in lamina propria and crypts from biopsies taken from 13 patients before start of low dose Naltrexone and 5 patients 3 months into treatment. Lower levels of GRP78 expression were observed in lamina propria, although this did not reach statistical significance. **c** Paired biopsies were available from three patients. All three showed reduction of GRP78 expression in the lamina propria upon treatment with low dose Naltrexone. **d** Two paired samples are shown. Patient A was a non-responder, Patient B did show clinical response to low dose Naltrexone

Next, we investigated intestinal ER stress in patients treated with LDN. Intestinal tissue biopsies were available in 13 patients prior to treatment and in 5 patients 3 months into treatment, with 3 paired samples. Sections were stained for GRP78 (for specificity of the staining, see Additional file 4: Figure S3). High levels of ER stress were observed in both the inflamed intestinal lamina propria and crypts from IBD patients (Fig. 5a). GRP78 levels decreased upon NTX treatment, most noticeably in the lamina propria, (1.14 ± 0.5 vs 0.8 ± 0.5 for lamina propria and 0.9 ± 0.4 vs 0.7 ± 0.6 for crypts) although statistical significance was not reached because of low patient numbers (Fig. 4b). However, in the 3 paired samples available, NTX treatment reduced interstitial ER stress (Fig. 4c, and examples in Fig. 4d). In toto, these data suggest that Naltrexone has a direct effect on ER stress as measured by GRP78 expression in intestinal mucosa.

## Discussion

In this study, therapy refractory IBD patients receiving LDN showed clinical improvement in 74.5% of all patients and long-lasting clinical remission of in 25.5%. Furthermore, most patients achieving clinical remission also showed endoscopic improvement. The response and remission rates in this study appear slightly lower than the rates found in previously published studies (response rates of 88–89% and remission rates of 30–67% [22, 23]). These differences might be explained by differences in patient population, as the patients in our cohort had more severe disease, as reflected by the differences in previous drug exposure. Furthermore, the sample size of the previous studies was small, with only 17 and 18 patients receiving Naltrexone in the pilot study and the placebo controlled study, respectively. No serious adverse events were reported in the current study. Interestingly, we also found no elevated liver enzymes in our cohort, whereas previous studies found such abnormalities in 1.8–11.1% of patients treated with Naltrexone [22, 23]. Thus, our data suggest that LDN is safe and effective in the treatment of conventional therapy-refractory IBD patients.

While the potential benefit of Naltrexone treatment for IBD is becoming clear, the underlying mechanisms and the general role of the opioid system in IBD have so far received very little attention. An increased expression of MOR in mucosal immune cells has been shown, and one possible function of this upregulation may be compensatory pain management. Pro-inflammatory Th1 and Th17 cells produce enhanced levels of endogenous opioids during colitis in mice [31], which suppress pain signals during chronic mucosal inflammation [32]. As such, it is conceivable that part of the remission in LDN treated patients is a result of a general improvement of well-being. Interestingly, antagonists of the nociceptor receptor (involved in pain sensation) also reduced intestinal pro-inflammatory cytokine profiles and ameliorated DSS colitis in mice, suggesting that blocking pain sensors has a direct immune-modulatory effect [33]. Intriguingly, it has recently been shown that the opioid inactive (+)-isomers of Naltrexone inhibit lipopolysaccharide-induced Toll like Receptor 4 (TLR4) signaling, a bacterial-induced inflammatory pathway contributing to IBD [34, 35]. It is as yet unclear whether the Naltrexone preparations currently used in patients (and as bought for in vitro experiments) contain this opioid inactive isoform, but it is at least theoretically possible that some of the beneficial effects observed in the current study are not regulated by MOR, but rather by inhibition of TLR signaling. Furthermore, in addition to MOR, Naltrexone also has weak affinity for the κ and δ opioid receptors, and it is conceivable that some of the observed effects occur through these receptors.

The limited studies performed so far on the mechanistic effect of Naltrexone have mainly focused on immune cells. However, our study suggests that Naltrexone can also have direct beneficial consequences on epithelial barrier cells, by stimulating wound healing. These data are in accordance with the improved in vivo wound healing observed upon Naltrexone treatment in both IBD patients and diabetic mice [36]. However, while the effect of Naltrexone on wound healing in skin was shown to be a result of increased fibroblast proliferation [37], our in vitro model suggests that wound healing of intestinal epithelial barriers is modulated by improved migration rather than proliferation.

Other studies investigating the potential mechanism of LDN on inflammation have focused on immune cell cytokine production. Elevated TNFα, IL-6 and IL-12 levels have been reported to be reduced by Naltrexone in chemically induced mouse colitis models [15, 16]. In contrast, others have found that LDN enhances dendritic cell maturation and stimulates their TNFα and IL-12 production, whereas in the current study, no effect of Naltrexone on either epithelial induced IL-8 production or IL-8 and TNFα serum levels in IBD patients was observed. However, it should be noted that not all cytokines could be detected in our system, and it is possible that other cytokines, which were not studied here, are affected.

We and others have previously shown that patients carrying gene variants associated with development of IBD demonstrate increased mucosal ER stress and bacterial persistence, suggesting that intestinal ER stress contributes to IBD pathology [19, 38–40]. The cell type that appeared most affected, even in non-inflamed mucosa, were the Paneth cells, specialized anti-microbial peptide producing cells [17]. We now show that during inflammation, not only Paneth cells, but also other crypt and lamina propria cells show increased ER stress, which may reflect a general cellular stress response in the presence of pro-inflammatory cytokines or bacteria. Indeed, we demonstrate that stimulation of intestinal epithelial cells with bacteria or LPS triggers a significant upregulation of the ER stress marker GRP78. However, not all cell lines showed this effect, which may be a reflection of the genetic IBD risk factors present in these cell lines. Nevertheless, ER stress in both cell lines as well as organoids derived from IBD patients was reduced by treatment with Naltrexone, as were lamina propria GRP78 levels in biopsies from patients treated with Naltrexone, although this did not correlate with clinical response in all cases. Interestingly, genetic variants of the MOR gene *OPRM1* affect response to high doses of NTX, however to what extent they may play a role in clinical and molecular response in IBD patients is as yet unclear [41].

## Conclusion

In conclusion, our study provides additional insight into the mechanism of action of Naltrexone in intestinal inflammation, showing a direct effect of this opioid on intestinal epithelial wound healing and ER stress reduction. The clinical results are promising, and particularly given the low frequency and relative beneficial nature of side-effects, the use of LDN in therapy refractory IBD patients seems warranted. Future clinical research may also focus on the use of LDN earlier in the IBD treatment pyramid.

## Additional files

**Additional file 1.** Additional data.

**Additional file 2: Figure S1.** Naltrexone does not affect cell viability at concentrations up to 100 μM Naltrexone. Cell viability as determined by MTT assays after 24 h **(A),** 48 h **(B)** and 72 h **(C)** of incubation with increasing concentrations of Naltrexone (NTX). Mean of two independent experiments is shown.

**Additional file 3: Figure S2.** **(A)** ER stress was induced in CACO2 cells by treatment with 2 μM Tunicamycin (Tuni), resulting in an upregulation of GRP78 expression levels as detected by Western Blot analysis. Co-treatment of cells with 1 μg/mL Naltrexone (NTX) reduces the amount of Tunicamycin-induced GRP78 expression. Upper graph: mean densitometry values of two independent experiments, GRP78 expression is corrected for Actin, to control for equal loading. Representative example is shown in the bottom panels. **(B)** Treatment of CACO2 cells with bacteria does not affect GRP78 as much as in HCT116 cells. Mean densitometry values of threer independent experiments is shown. **(C)** Treatment of CACO2 cells with LPS mildly upregulates CHOP expression as detected by Western blot analysis, which is reduced by co-treatment cells by treatment of cells with 1 μg/mL Naltrexone. Mean densitometry values of one experiments is shown.

**Additional file 4: Figure S3.** GRP78 staining specificity. A patient with no GRP78 expression (left panel) and a patient showing clusters of GRP78 positivity alongside negative tissue (right panel) are shown.

## Abbreviations

anti-TNF: anti-tumor necrosis factor
CD: Crohn’s disease
CDEIS: Crohn’s disease endoscopic index of severity
CRP: C-reactive protein
IBD: inflammatory bowel disease
IQR: interquartile range
LDN: low dose Naltrexone
NTX: Naltrexone
SES-CD: simple endoscopic score for Crohn’s disease
UC: ulcerative colitis
