# Supplementary material for: Low dose Naltrexone for induction of remission in inflammatory bowel disease patients
Source: J Transl Med. 2018 Mar 9;16:55. doi: 10.1186/s12967-018-1427-5 (PMC5845217; doi:10.1186/s12967-018-1427-5)
Supplement: Supplementary file 1 — Additional file 1. Additional data. [file 12967_2018_1427_MOESM1_ESM.docx]

Additional data to:

**Low dose Naltrexone for induction of remission in inflammatory bowel disease patients**

Mitchell RKL Lie^*^, Janine van der Giessen^*^, Gwenny M Fuhler^*^, Alison de Lima, Maikel P Peppelenbosch, Cokkie van der Ent, C. Janneke van der Woude^#^

Erasmus MC - University Medical Centre Rotterdam, Department of Gastroenterology and Hepatology

Material and Methods:

Organoid culture

Non-inflamed intestinal biopsies were collected from two IBD patients undergoing routine endoscopy for their disease. Organoids were collected in PBS and transferred into a 15 mL tube containing 10 mL complete chelating solution (CCS, MilliQ H_2_O was supplemented with 1.0 g/L of Na2HPO4-2H2O, 1.08 g/L of KH2PO4, 5.6 g/L of NaCl, 0.12 g/L of KCl, 15 g/L of Sucrose, 10 g/L of D-Sorbitol and 80 lg/L of DL-dithiothreitol). Biopsies were washed three times by pipetting up and down 8–10 times. Biopsies were transferred into a 50 mL tube with 5mL CCS and 100 uL 0.5M EDTA was added, then incubated on a rollerplate for 35 minutes at 4°C. Supernatant with EDTA was discarded and 5 ml fresh CCS solution was added. It was thoroughly suspended by pipetting up and down with 10 ml tip for 8–10 times to loosen crypts. Supernatant with crypts was transferred into a 15 ml tube and 2 ml FCS was added. Then, crypt suspension was centrifuged at 1200 RPM for 5 min. Supernatant was discarded and crypts were re-suspended in 12 ml cold advanced DMEM (Advanced DMEM/F12, 5 mL 100x GlutaMAX (GMX), 1% P/S, 500 μL Gentamicin and 5mL 1M HEPES) and centrifuged at 800 RPM for 5 min at 4 °C. Crypts were suspended in 50 μL growth factor reduced phenol-red free Matrigel (Corning, Bedford, USA). Then, a 50 μL droplet of Matrigel/crypt mix was placed in the center of each well of a 24-well plate, and was subsequently incubated at 37°C with 5% CO2 for 15 min. 700 μL of culture medium was added per well. The culture medium was supplemented with CMGF-, 2% of B-27 supplements (Gibco, Grand Island, USA), 1% of N2 Supplements (Gibco, Grand Island, USA), 500 pg/L of EGF, 1 mM n-Acetyl Cysteine, 10 mM Nicotinamide, 0.5 μM A83-01 (TGF-b inhibitor), 3 μM SB202190 (p38 inhibitor), 20% (vol/vol) of R-Spondin 1 (conditioned medium), 10% (vol/vol) of Noggin (conditioned medium) and 50% (vol/vol) of Wnt3a (conditioned medium). Culture medium was refreshed every 3 days, and organoids were passaged every 7 days. Passaging of human organoids was done by solubilizing Matrigel and mechanically breaking up the organoids by passing through a 5 ml tip inserting a 200 μL tip, single cells were then transferred to fresh Matrigel. The passaging was performed every 5–6 days with a 1:3 split ratio. Each well contains 10 or more organoids.

Cell viability assay

Cell viability was assessed using MTT assays. Cells (10,000) were seeded in 96 wells plates, and upon adhesion were treated with different concentrations of Naltrexone (Sigma Aldrich, St Louis, MA). After 24h 48h and 72h, cells were incubated with 5mM MTT (3-(4,5-Dimethylthiazol-2-yl)-2,5-diphenyltetrazolium bromide, Sigma Aldrich, St Louis, MA) for 3h and colorimetric changes were measured using a microplate reader (Model 680XR Bio-Rad) at 490 and 595 nm.

Wound healing assay

In short, in scratch-wound assays, cell monolayers were scratched with a pipette tip, washed twice, and treated with 1 μM Naltrexone or vehicle control. Concentration Naltrexone used was based on *in vivo* dosages (4.5 mg per ±60 kg bodyweight). Photographs were taken (Axiovert200 M microscope; Carl Zeiss BV, Sliedrecht, The Netherlands) to analyze the percentage of open wound area at 24 h (ImageJ software; US National Institutes of Health, Bethesda, MD, USA). Experiments were performed thrice in duplicate, with two measure-sites per scratch.

## Western blotting

Western blotting was performed as described [26], with modifications. HCT116 and CACO-2 cells were treated with Tunicamycin (2μM) or *E. coli* (paraffin-fixed DH5α, 6.25e5/mL) or Lipopolysaccharides (LPS, 10μg/mL) in the presence or absence of 1 μg/mL Naltrexone. Organoids were treated with LPS (10μg/mL) in the presence or absence of 1 μg/mL Naltrexone. Subsequently, cells were washed with PBS and lysed on ice in 300µL 2x concentrated Laemmli buffer (100mM Tris–HCl (pH 6.8), 200mM dithiothreitol, 4% SDS, 0.1% bromophenol blue, 20% glycerol, and 2% DTT) and boiled for 5 minutes at 95°C. Organoids were also washed with PBS, matrigel was dissolved and the organoids were released. This solution was transferred to a tube and centrifuged for 8 minutes at 800 RPM. Supernatant was removed and the pellet was resuspended in 150μL Laemli buffer and boiled for 5 minutes at 95°C.

Cell extracts were resolved by SDS–PAGE and transferred to polyvinylidene difluoride membranes (Merck chemicals BV, Amsterdam, the Netherlands). Membranes were blocked in 50% odyssey blocking buffer (LI-COR Biosciences, Lincoln, NE) in PBS/0.05% Tween-20 and incubated overnight at 4°C with primary antibody. After washing in PBS-T, membranes were incubated with IRDye® antibodies (LI-COR Biosciences, Lincoln, NE) for 1h. Detection was performed using Odyssey reader and analyzed using manufacturers software. Experiments were performed at least twice.

## Immunohistochemistry

FFPE tissue sections were immunohistologically stained for GRP78. Briefly, 5µm sections were deparaffinized in xylene and rinsed through graded alcohols (100% alcohols (18:1:1 100% ethanol: 100% methanol: 100% isopropanol), a 95% solution of the 100% alcohols, and a 80% solution of the 100% alcohols). Next, slides were rinsed several times with fresh deionized water, followed by another 5 minutes wash using fresh water. Antigen retrieval was performed by boiling the slides in 600mL of 10mM sodium citrate buffer, pH 6.0 for 15 minutes. Slides were cooled for 20 minutes and washed extensively in double-distilled H_2_O and PBS. Endogenous peroxides were blocked by soaking slides in a PBS/3% H_2_O_2_ solution for 10 minutes at room temperature. Subsequently, slides were rinsed in PBS and blocked by incubating in 10% goat serum in PBS at room temperature for 1h. Thereafter, tissue sections were incubated with GRP78 antibody (BiP, Cell Signaling Technology, Danvers, MA) diluted in blocking buffer (1:100) overnight at 4°C. Next, slides were rinsed again in PBS for 5 minutes each wash. Rabbit envision (DAKO, Heverlee, Belgium) was used as secondary antibody.

Reverse transcriptase polymerase chain reaction (rt-PCR)

We used rt-PCR to determine MOR expression on the IEC cell lines. RNA was isolated as described previously [29]. Briefly, RNA was isolated using a NucleoSpin® RNA kit (MACHEREY-NAGEL, Düren, Germany) and cDNA was synthesized using the TAKARA reverse transcription system (TAKARA BIO INC, Shiga, Japan) PCR was performed in a 25μL reaction, using GoTaq polymerase and GoTaq Flexi buffer, 2 mM MgCl2 (Promega, Madison, WI), dNTP (0.5mM each, Roche), 50 ng template and 0.5nM of the following primers: Forward: 5’-GGAAGCCCTCCAGGTTCATT, Reverse 5’- GGTCTCTTCACTGGGCACTC. Ribosomal protein (*RP2*) primers were used as control: 5’-AAGCTGAGGATGCTCAAAGG, 5’-CCCATTAAACTCCAAGGCAA.
