# Supplementary figures and images for: Low dose Naltrexone for induction of remission in inflammatory bowel disease patients
Source: J Transl Med. 2018 Mar 9;16:55. doi: 10.1186/s12967-018-1427-5 (PMC5845217; doi:10.1186/s12967-018-1427-5)

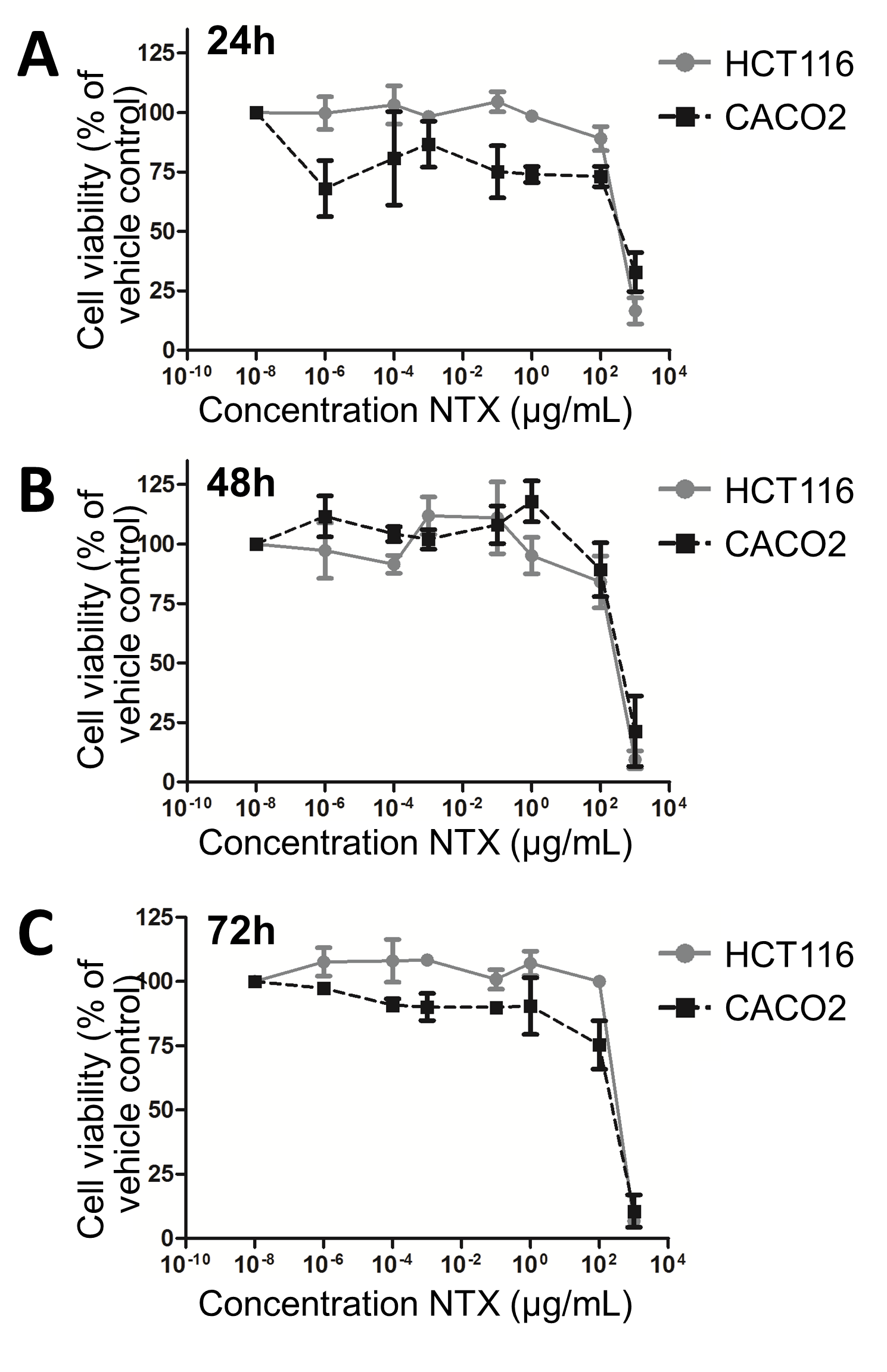

Supplement: Supplementary file 2 — Additional file 2: Figure S1. Naltrexone does not affect cell viability at concentrations up to 100 μM Naltrexone. Cell viability as determined by MTT assays after 24 h (A), 48 h (B) and 72 h (C) of incubation with increasing concentrations of Naltrexone (NTX). Mean of two independent experiments is shown. [file 12967_2018_1427_MOESM2_ESM.tif]

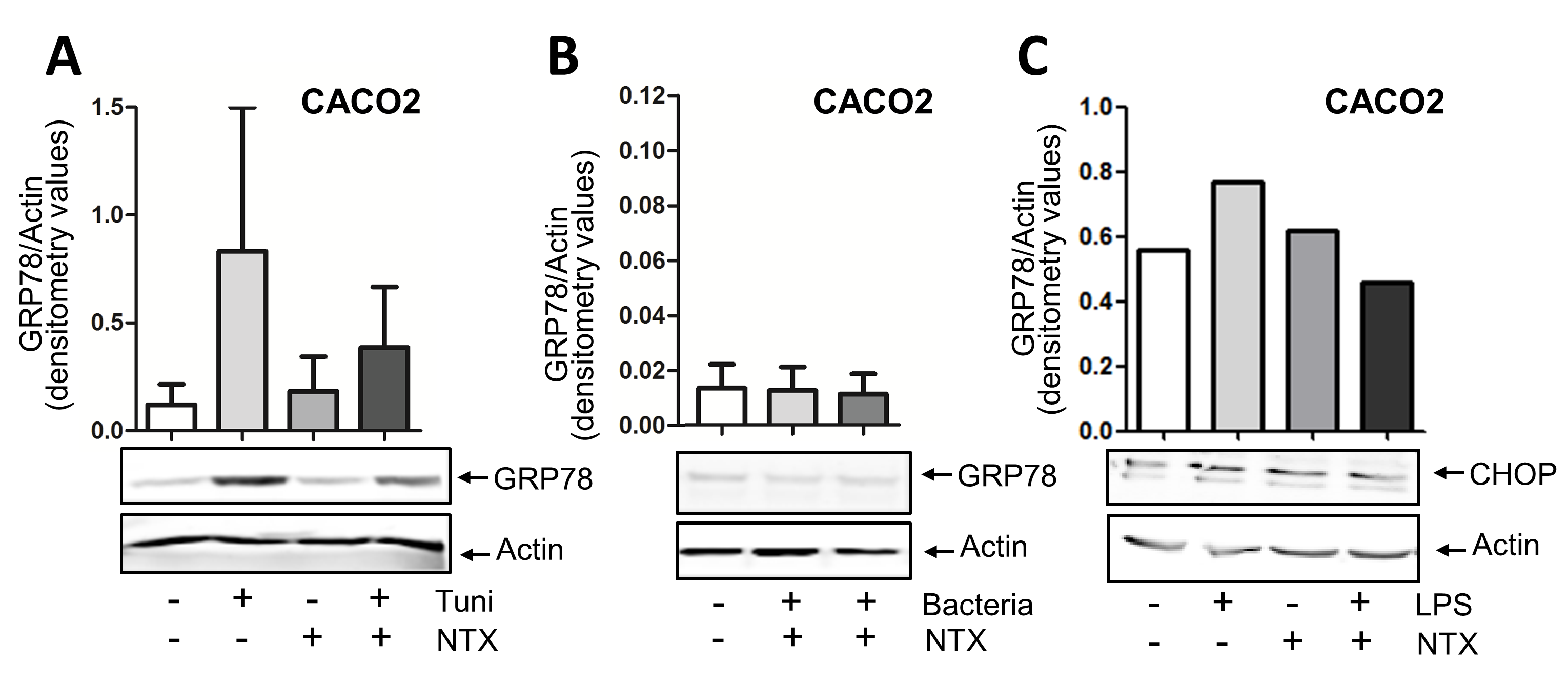

Supplement: Supplementary file 3 — Additional file 3: Figure S2. (A) ER stress was induced in CACO2 cells by treatment with 2 μM Tunicamycin (Tuni), resulting in an upregulation of GRP78 expression levels as detected by Western Blot analysis. Co-treatment of cells with 1 μg/mL Naltrexone (NTX) reduces the amount of Tunicamycin-induced GRP78 expression. Upper graph: mean densitometry values of two independent experiments, GRP78 expression is corrected for Actin, to control for equal loading. Representative example is shown in the bottom panels. (B) Treatment of CACO2 cells with bacteria does not affect GRP78 as much as in HCT116 cells. Mean densitometry values of threer independent experiments is shown. (C) Treatment of CACO2 cells with LPS mildly upregulates CHOP expression as detected by Western blot analysis, which is reduced by co-treatment cells by treatment of cells with 1 μg/mL Naltrexone. Mean densitometry values of one experiments is shown. [file 12967_2018_1427_MOESM3_ESM.tif]

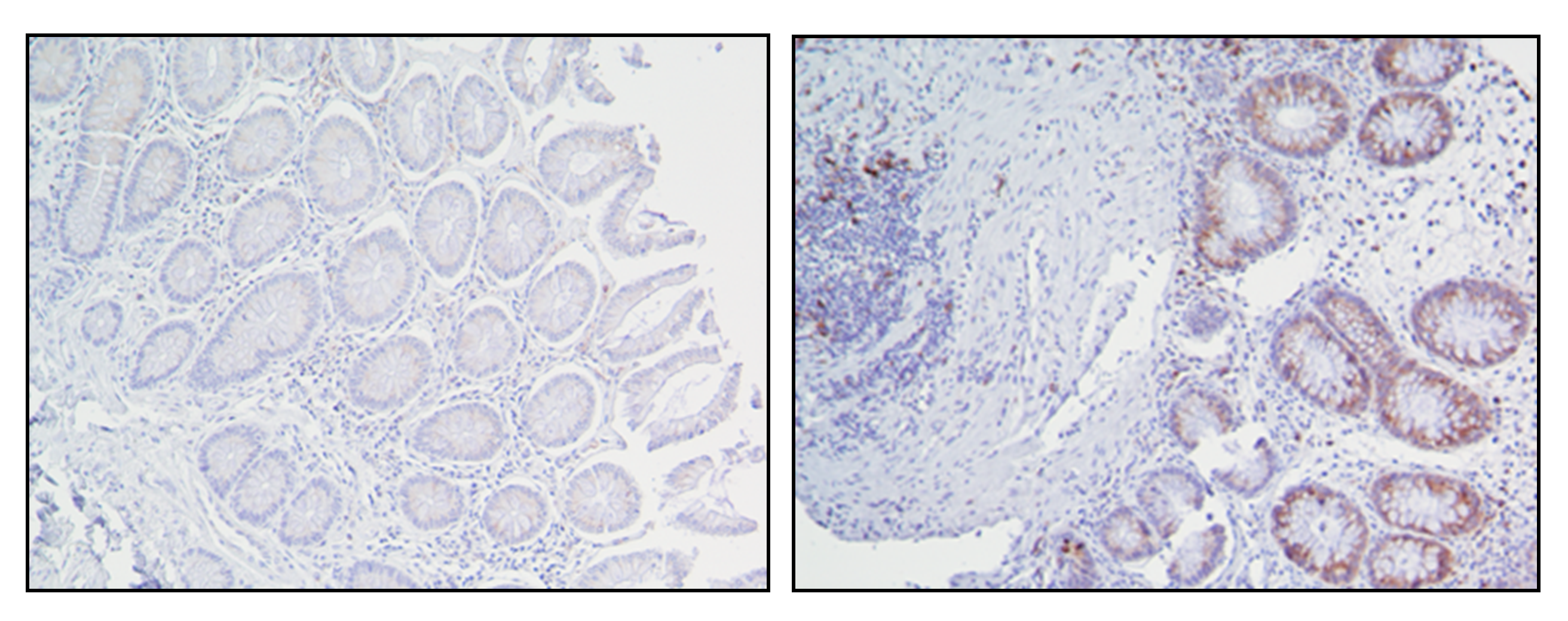

Supplement: Supplementary file 4 — Additional file 4: Figure S3. GRP78 staining specificity. A patient with no GRP78 expression (left panel) and a patient showing clusters of GRP78 positivity alongside negative tissue (right panel) are shown. [file 12967_2018_1427_MOESM4_ESM.tif]
